# Effect of Ni on Microstructure and Mechanical Property of a Co-Ti-V-Based Superalloy

**DOI:** 10.1155/2021/6678085

**Published:** 2021-05-26

**Authors:** Pengjie Zhou, Xinkang Gao, Dehang Song, Yinbing Liu, Jun Cheng

**Affiliations:** ^1^School of Materials Science and Engineering, Jiangsu University of Science and Technology, Zhenjiang 212003, China; ^2^Northwest Institute for Non-Ferrous Metal Research, Shanxi Key Laboratory of Biomedical Metal Materials, Xi'an 710016, China; ^3^State Key Laboratory of Solidification Processing, Northwestern Polytechnical University, Xi'an 710072, China

## Abstract

The effect of Ni on microstructure, elemental partition behavior, *γ*′ phase solvus temperature, lattice misfit between *γ* and *γ*′ phases, and mechanical properties of the Co-8Ti-11V-xNi alloys was investigated. The result shows that the lattice misfit in the alloys decreases from 0.74% to 0.61% as the Ni content increases from 0 to 10%, and the average sizes of the cuboidal *γ*′ phase were measured to be 312.10 nm, 112.86 nm, and 141.84 nm for the Co-8Ti-11V, Co-8Ti-11V-5Ni, and Co-8Ti-11V-10Ni, respectively. Ti, V, and Ni exhibit a slight tendency to partition into the *γ*′ phase, while Co shows a slight tendency to partition into the *γ* phase. The solvus temperatures of the *γ*′ phase were measured to be 1167°C, 1114°C, and 1108°C for the Co-8Ti-11V, Co-8Ti-11V-5Ni, and Co-8Ti-11V-10Ni alloys, respectively, by using differential scanning calorimetry (DSC). Moreover, the yield strength and ultimate strength of the Co-8Ti-11V, Co-8Ti-11V-5Ni, and Co-8Ti-11V-10Ni alloys were investigated, and the yield strength and ultimate strength of the 10Ni alloy were highest, at 219 MPa and 240 MPa. After compression at 1000°C, the dislocations cannot effectively shear the *γ*′ phase in the 0Ni and 10Ni alloys, resulting in a relatively high compressive strength of the 0Ni and 10Ni alloys. However, the *γ*′ phase of the 5Ni alloy is no longer visible, and its strength is the lowest.

## 1. Introduction

The superalloys, which are normally used in the industrial environments with elevated temperature and high pressure, can be divided into three classes named as nickel-based, cobalt-based, and iron-based superalloys [1, 2]. Among various types of superalloys, the nickel-based superalloys reinforced by the coherent ordered L12 structure phase are more widely utilized in modern industries than the other two types [3–5]. However, the carbide-strengthened Co-based superalloys exhibit higher incipient melting temperature and better corrosion resistance than the Ni-based superalloys. But it is believed that the lack of GCP phases in traditional carbide-strengthened Co-based superalloys leads to their inferior high-temperature strengths [6–13].

In 2006, Sato et al. reported *γ*/*γ*′-strengthened Co-Al-W-based superalloys, which possess some outstanding properties [14]. The discovery of the Co-Al-W-based superalloy indicated a promising candidate for high-temperature applications. However, some researchers found the *γ*′ phase in the Co-Al-W ternary alloy was metastable [15]. It needs to incorporate some alloying elements like Ni, Ti, B, and Cr to improve thermal stability [16–18]. The *γ*′-Co3(Al, W) in the ternary Co-Al-W system has a relatively low solvus temperature [19]. Other research tried to increase the volume fraction of the *γ*′ phase by increasing the contents of W element, resulting in an increase in the mass density of the alloys [20–22]. Since then, most research studies have focused on replacing some of the W with various refractory elements, such as Ta and Nb [14, 23], which can stabilize the *γ*′ phase, increase the *γ*′ phase solvus temperature, and improve the high temperature strength. Also, the Ni element was added to the Co-Al-W ternary system, which has been found to stabilize the *γ*′ phase [24]. Following the discovery that the *γ*/*γ*′ two-phase structure of the Co-Al-Mo-Nb alloy by Makineni et al. [25–28], some research studies have focused on W-free Co-based superalloys with *γ*/*γ*′ strengthening. The L12-ordered phases were discovered in other Co-based superalloys, such as Co-V, Co-Ti, and Co-Ta systems [29–32]. However, among these Co-based systems, only the *γ*′ phase in the Co-Ti system is considerably stable [33]. In the past, some ternary systems have been identified to exhibit ordered L12 structure *γ*′ phase, such as the Co-Ti-Cr ternary system and Co-Ti-V ternary system [34, 35]. The mass density of Co-Ti-Cr alloys hardened by the *γ*′ phase is about 14% lower than that of a typical Co-Al-W alloy and has a small lattice misfit between *γ* and *γ*′ and a higher *γ*′ phase volume fraction [34]. Recently, Ruan et al. reported the effects of Al and Ni on the microstructure, phase stability, and high-temperature mechanical properties of Co-Ti-V alloys [36].

In this study, three Co-Ti-V alloys with different Ni contents were prepared, and the effects of Ni in microstructure, elemental partition behavior, lattice misfits between *γ* and *γ*′ phases, and mechanical properties of the Co-Ti-V-based superalloys were investigated.

## 2. Materials and Methods

Experimental alloys with 80 g in weight were prepared from high-purity Co (99.9 wt %), Ti (99.9 wt %), V (99.95 wt %), and Ru (99.9 wt %) in a vacuum arc furnace. The cast ingots were turned over and remelted 5 times to achieve the composition homogeneity. Then, the experimental alloys were put into quartz capsules and filled with argon gas. The nominal compositions of the three experimental alloys are listed in Table 1. In this study, there are three experimental alloys defined as 0Ni, 5Ni, and 10Ni, respectively.

The cast materials were solutionized at 1100°C for 48 h and aged at 870°C for 72 h under an argon atmosphere. To obtain the best mechanical properties and investigate the morphologies of the *γ*′ phase, the samples were quickly quenched into ice water after the heat treatment. The microstructural morphology was investigated by using scanning electron microscopy (SEM) (Zeiss Merlin Compact), which was operated at 10 kV. The samples were mechanically polished and then electroetched in a solution of HNO3 (vol. 5%)+CH3COOH (vol. 15%)+distilled water (vol. 85%) for a few seconds before observations. The Image-Pro Plus 6.0 software was used to calculate the average sizes of the *γ*′ phase. The *γ*/*γ*′ two-phase lattice misfits were investigated by X-ray diffraction (XRD-6000) analysis, using Cu K*α* radiation at 40 kV and 40 mA. The tested powders were machined from bulk materials. The curves were fitted by using the data analysis and technical graphing software Origin Pro 9.1. Details of the microstructure and partitioning behavior of each element were obtained by using a transmission electron microscope (JEM-2100F TEM) equipped with the Oxford-80T energy spectrometer (EDS). Thin foil specimens for TEM analyses were prepared by twin jet electropolishing in a solution of HClO_4_ (vol. 5%)+CH_3_CH_2_OH (vol. 95%), and the voltage was 19 V and the temperature was -30°C, respectively.

Besides, the high-temperature compression tests were performed through a Gleeble-3800 thermal simulator, which is equipped with the strain rate of 3 × 10^−4^ s^−1^ and a heating rate of 10°C/s at 1000°C. The compression samples were made the shape with a *Φ*6 mm × 9 mm rod by wire cutting. The solvus temperature of the *γ*′ phase was examined though differential scanning calorimetry (DSC) with an argon atmosphere at a heating rate of 20°C/min.

## 3. Results and Discussions

### 3.1. Morphologies of the *γ*′ Phase

**Figure 1:**
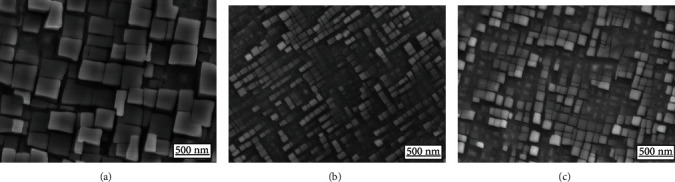
SEM micrograph of the (a) 0Ni alloy, (b) 5Ni alloy, and (c) 10Ni alloy, after solution treatment for 48 h at 1100°C followed by aging treatment for 72 h at 870°C. The samples were electroetched.

Figure 1 shows scanning electron microscopy (SEM) micrographs for ternary Co-8Ti-11V, quaternary Co-8Ti-11V-5Ni, and quaternary Co-8Ti-11V-10Ni, which indicates the presence of cuboidal *γ*′-L12 precipitates after being electroetched. The SEM micrograph of the 0Ni alloy is shown in Figure 1(a), which possesses large size and high volume fraction of the *γ*′ phase.  Figure 1(b) is a SEM micrograph of the 5Ni alloy, showing a uniform cuboidal shape of the *γ*′ phase, and the size of the *γ*′ precipitates was distributed in a large scatter. It is obvious that the coarsening behavior of the *γ*′ phase was restrained by the adding of 5%Ni, and the 5% Ni alloy possesses smaller size and low volume fraction of the *γ*′ phase. A more uniform cuboidal shape of the *γ*′ phase is shown in Figure 1(c). The average sizes of the *γ*′ phase were measured to be 312.10 nm, 112.86 nm, and 141.84 nm for the 0Ni, 5Ni, and 10Ni alloys, respectively, by using the Image-Pro Plus 6.0 software. The results indicate that adding Ni content can retard the coarsening behavior of the *γ*′ phase. Meher et al. [37] measured the coarsening behavior of Co-10Al-10W at 800°C. Pandey et al. [38] and Wang et al. [39] also reported that the coarsening behavior of the *γ*′ phases was limited by adding Re.

### 3.2. The Lattice Misfit between *γ* and *γ*′ Phases

**Figure 2:**
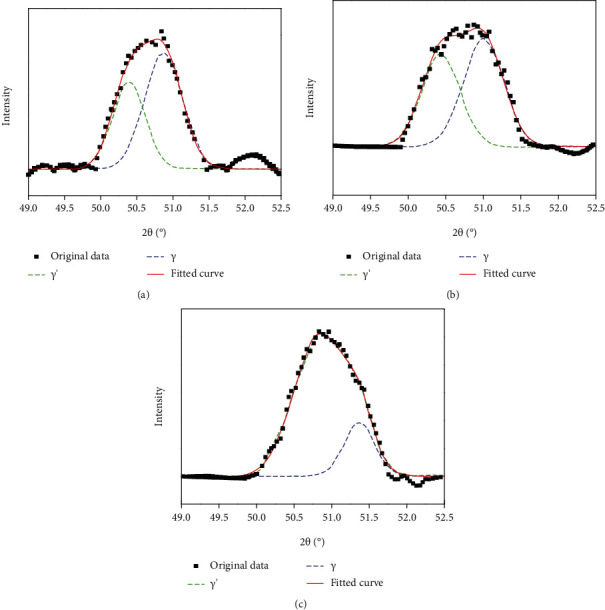
The X-ray diffraction (200) peaks obtained from (a) 0Ni alloy, (b) 5Ni alloy, and (c) 10Ni alloy aging treatment for 72 h at 870°C after solution treatment for 48 h at 1100°C.

The lattice misfit between *γ* and *γ*′ phases was calculated by X-ray diffraction analysis with different components of alloys. The data were measured in the range of 2*θ* from 49° to 52.5°, and a scanning speed of 0.125°/min was selected for XRD testing.  Figure 2 shows the X-ray diffraction (200) peaks of the 0Ni, 5Ni, and 10Ni alloys. The lattice constant of *γ* and *γ*′ and lattice misfit between *γ* and *γ*′ in the alloys 0Ni, 5Ni, and 10Ni were measured, which are shown in Table 2.

As can be seen from Figure 2, compared with the 0Ni alloy, the diffraction peak of the *γ*′ phase of the 5Ni alloy was slightly right shifted, indicating that the lattice constant of the *γ*′ phase in the 5Ni alloy dropped, which decreases the lattice constant gap between the *γ* and *γ*′ phases of the 5Ni alloy. Compared with the other two alloys, the diffraction peak of the *γ*′ phase of the 10Ni alloy was further right, thus further decreasing the lattice constant of the *γ*′ phase of the 10Ni alloy, and the lattice constant gap between *γ* and *γ*′ also decreased.

For the 0Ni alloy, the lattice constant of the *γ*′ phase is 0.3611 nm and the lattice constant of the *γ* phase is 0.3584 nm, so the lattice misfit is 0.74%. When the Ni content increases to 5%, the lattice constants of *γ*′ and *γ* in the 5Ni alloy are 0.3607 nm and 0.3583 nm, respectively. So the lattice misfit between *γ* and *γ*′ phases is 0.68%. Compared to the 0Ni alloy, the lattice misfit in the 5Ni alloy decreases. The lattice constants between *γ*′ and *γ* phases of the 10Ni alloy are 0.3593 nm and 0.3571 nm, respectively, and the lattice misfit between *γ* and *γ*′ phases is 0.61%. Compared to the 0Ni and 5Ni alloys, the lattice misfit in the 10Ni alloy decreased. The result shows that the lattice misfit between *γ* and *γ*′ phases decreases with increasing Ni content.

The lattice parameter misfit of Co-Ti-1Re, Co-Ti-3Re, and Co-Ti-5Re alloys when aged at 800°C for 24 h were 72%, 0.58%, and 0.50%, respectively [40]. The lattice misfit between *γ* and *γ*′ phases of Co-9.2Al-9W-based superalloys was 0.53% [14], while this study measures the lattice misfit of the Co-8Ti-11V alloy which is 0.77%. There is a large lattice misfit between the *γ* phase and the *γ*′-Co_3_Ti phase of the alloys reinforced by *γ*′-Co_3_Ti [41, 42]. Shinagawa et al. reported that as the Ni contents increase from 10% to 60%, the lattice misfit between *γ* and *γ*′ phases decreases [43]. Besides, the atomic radius of Ti is greater than that of the Al and W elements, so the lattice constant of the *γ*′ phase increases, resulting in an increase in the lattice misfit.

### 3.3. Microstructure and Partitioning Behavior

**Figure 3:**
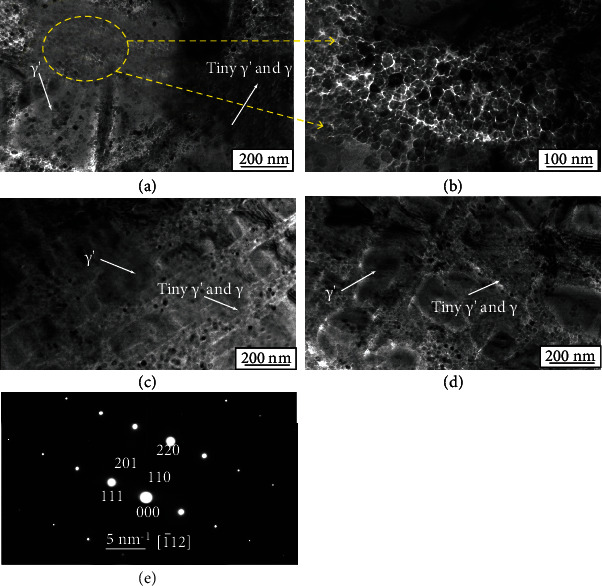
Bright-field TEM images of the (a, b) 0Ni alloy, (c) 5Ni alloy, and (d) 10Ni alloy and (e) SADP of cubical *γ*′ in 5Ni alloy aging treatment for 72 h at 870°C after solution treatment for 48 h at 1100°C.

Figure 3 shows bright-field TEM images of the 0Ni, 5Ni, and 10Ni alloys. The dual size of the *γ*′ precipitates is observed in Figures 3(a)–3(d), respectively. The larger *γ*′ precipitates are cubical or trigonal in shape, and the fine *γ*′ precipitates are spherical particles distributed in the *γ* matrix channels. They are distributed throughout the *γ* matrix homogeneously. A selected area diffraction pattern (SADP) is shown in Figure 3(e), the superlattice diffraction spot of the (110) plane indicated an ordered L1_2_ structure, and the spherical particles of the precipitates were identified as the L1_2_-ordered *γ*′ phase (Co_3_Ti, PDF no. 23-0938). The average sizes of fine spherical *γ*′ precipitates were measured to be 18.09 nm, 16.95 nm, and 17.89 nm for the 0Ni, 5Ni, and 10Ni, respectively, by using the Image-Pro Plus 6.0 software. However, compared with the other two alloys, the 5Ni alloys possess low volume fraction of the *γ*′ phase.

**Figure 4:**
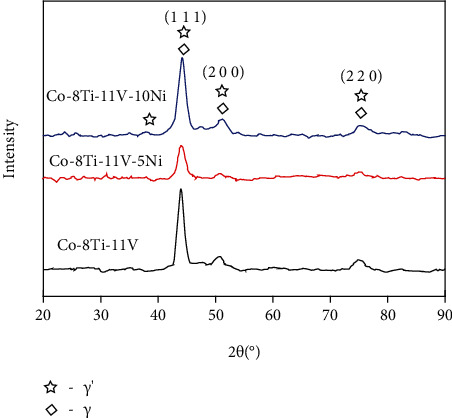
The XRD patterns of the 0Ni, 5Ni, and 10Ni alloys.

Figure 4 shows the XRD patterns of alloys with varied Ni contents. The XRD pattern of each alloy has three distinct peaks, in which crystal indices were identified as the (1 1 1), (2 0 0), and (2 2 0) planes, respectively. There is no other phase at the XRD patterns except for the typical two-phase microstructure *γ*′ phase and *γ* matrix phase.

The partitioning behaviors of the elements of the alloys with different components were analyzed by EDS equipped with TEM.  Table 3 shows the partitioning situation of the elements between *γ* and *γ*′ in the alloy. The partitioning coefficient *Kx* is defined as *Kx* = *C*_*γ*′−*x*_/*C*_*γ*−*x*_, where *C*_*γ*′−*x*_ and *C*_*γ*−*x*_ are the concentration of element X in the *γ*′ phase and the *γ* phase, respectively. Element X tends to partition into the *γ*′ phase when *Kx* > 1, while element X tends to partition into the *γ* phase when *Kx* < 1 [36].

**Figure 5:**
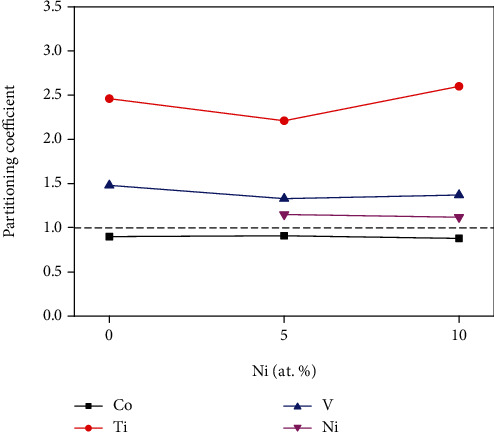
Partitioning coefficients of each element between *γ* and *γ*′ phases.

It can be calculated that the partitioning coefficient of the Co element was less than 1, so the Co element tends to partition into the *γ* phase more than into the *γ* phase. The partitioning coefficient of Ti, V, and Ni elements was greater than 1, tending to partition into the *γ*′ phase, and the Ti, V, and Ni elements were enriched in the *γ*′ phase. The partitioning coefficients of each element between *γ* and *γ*′ phases are shown in Figure 5. From the figure, the partitioning coefficient of the Co, V, and Ni element was relatively stable. When the Ni contents increase to 5%, the partitioning coefficients of the Ti element drop to a minimum. However, when the Ni contents increase to 10%, the partitioning coefficients of the Ti element increase. The results show that the Ti element partitioning into the *γ*′ phase was restrained for the 5Ni alloy and the Ti element partitioning into the *γ*′ phase was promoted for the 10Ni alloy. The alloying elements like Ti, Cr, Nb, Mo, Ta, W, and Ni were usually chosen for alloying additions in Ni-based and Co-based superalloys, due to the fact that they are often added to alloys for various properties such as *γ*′ phase stability and oxidation resistance [44–46].

**Figure 6:**
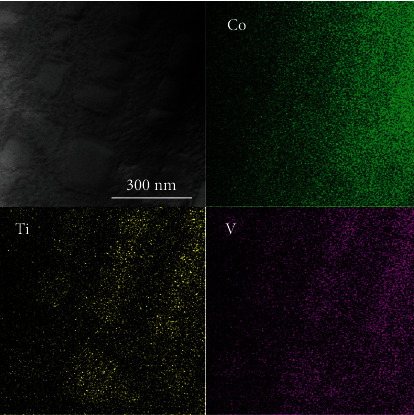
STEM image and elemental mappings for the *γ*′ cuboidal precipitates of the 0Ni alloy.

A TEM image and elemental mappings for the *γ*′ cuboidal precipitates of the 0Ni alloy are shown in Figure 6. As seen in Figure 6, Ti and V elements tend to be concentrated in the *γ*′ phase and enriched in the *γ*′ phase, whereas Co tends to partition to the *γ* matrix phase and is slightly enriched in the *γ* phase. Omori et al. reported that Ti, Nb, Mo, Ta, and W tend to partition into the *γ*′ phase, while the Cr element tends to partition to the *γ* phase, which is similar to that of Co-Al-W-based superalloys [46]. The Al element was distributed nearly uniformly in both *γ* and *γ*′ phases; however, the V element tends to partition into the *γ*′ phase, which is similar to that of Co-Al-W-X superalloys [46].

### 3.4. The Solvus Temperature of the *γ*′ Phase

**Figure 7:**
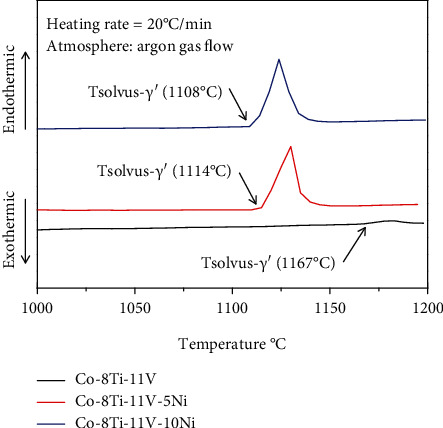
DSC heat curves of 0Ni, 5Ni, and 10Ni alloys.

Figure 7 shows the DSC heat curves of 0Ni, 5Ni, and 10Ni alloys, respectively. The *T*_solvus‐*γ*′_ means the solvus temperature of the *γ*′ phase. The *T*_solvus‐*γ*′_ of 0Ni, 5Ni, and 10Ni alloys was determined to be 1167°C, 1114°C, and 1108°C, respectively. The result shows that *T*_solvus‐*γ*′_ of alloys decreases as the Ni content increases. The solvus temperature of the *γ*′ phase in the Co-9.2Al-9W ternary system is 1263 K [14], and the solvus temperature of the *γ*′ phase in the Co-9Al-9W alloy is 1000°C [5]. The *T*_solvus‐*γ*′_ of alloys 0Ni, 5Ni, and 10Ni is higher that of Co-9.2Al-9W by 177°C, 124°C, and 118°C, respectively. The *T*_solvus−*γ*′_ of alloys 0Ni, 5Ni, and 10Ni is higher than that of Co-9Al-9W by 167°C, 114°C, and 108°C, respectively. The solvus temperature of the *γ*′ phase in the Co-5Ti-15V alloy is 1091°C [36]; the solvus temperature of the *γ*′ phase in the Co-8Ti-11V alloy is higher that of Co-5Ti-15V by 76°C. And Al is found to increase the *T*_solvus‐*γ*′_ by 21°C, but Ni is found to decrease the *T*_solvus‐*γ*′_ by 9°C [33]. The *T*_solvus‐*γ*′_ of the Ni-based superalloy IN-939 is 1100°C [47], while the *T*_solvus‐*γ*′_ of the Co-8Ti-11V alloy is higher than that of the Ni-based superalloy IN-939 by 67°C.

### 3.5. Mechanical Properties

**Figure 8:**
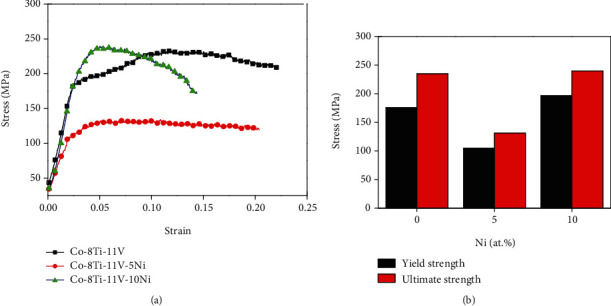
High-temperature compression curves and histogram of 0Ni, 5Ni, and 10Ni alloys of high-temperature compression tests at 1000°C.

Figure 8 shows the high-temperature compression curves and histogram of 0Ni, 5Ni, and 10Ni alloys after high-temperature compression tests at 1000°C. As can be seen in Figure 8, the yield strength and ultimate strength of the 10Ni alloy were at the maximum, at 219 MPa and 240 MPa, respectively. The yield strength and ultimate strength of the 0Ni alloy were 176 MPa and 235 MPa, respectively, which were similar to those of the Co-5Ti-15V alloy [36]. Compared with the other two alloys, the yield strength and ultimate strength of the 5Ni alloy were the smallest, which were only 105 MPa and 131 MPa, respectively. In general, there is a close relationship with the lattice misfit, the volume, and the size of the *γ*′ phase, the partitioning behavior of the elements in the alloy, and the high-temperature mechanical properties of the alloy [48, 49]. Adding *γ*′ phase stabilization elements to the alloy would increase the volume fraction of the *γ*′ phase [36], which improves the high-temperature mechanical properties of the alloy. In this study, the Ni element exhibits a slight tendency to partition into the *γ*′ phase and is enriched in the *γ*′ phase, so the mechanical properties of the 10Ni alloy were better than those of the other two alloys.

### 3.6. Compression Deformation

**Figure 9:**
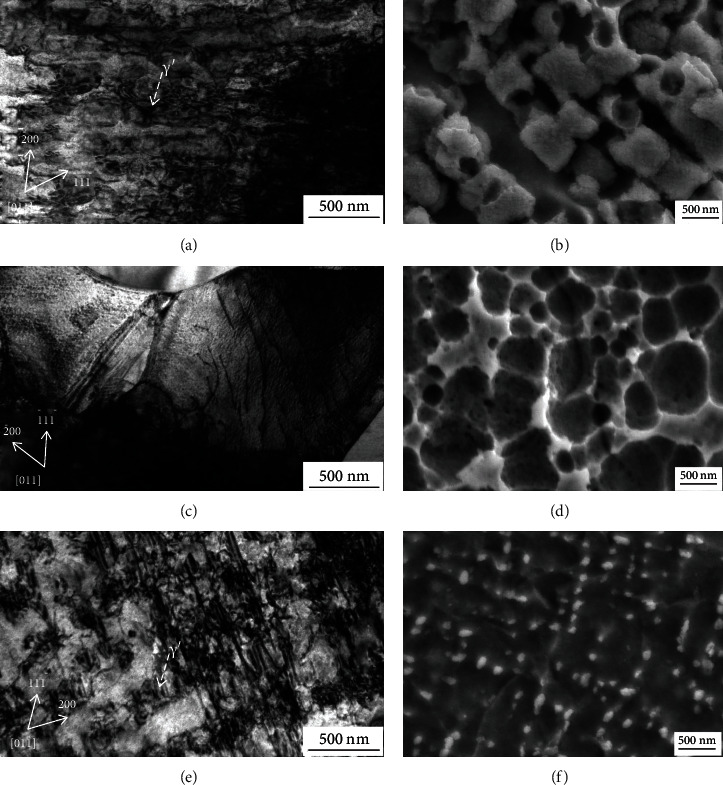
Bright-field TEM and SEM images of the (a, b) 0Ni alloy, (c, d) 5Ni alloy, and (e, f) 10Ni alloy after high-temperature compression tests at 1000°C.

There is a key relationship between dislocation deformation and mechanical properties of alloys.  Figure 9 shows the bright-field TEM and SEM images of the alloy with varied Ni contents after high-temperature compression tests. There are a considerable number of dislocations in the *γ*′ phase of the 0Ni alloy. The *γ*′ phase is sheared by the dislocations, as shown in Figure 9(a).  Figure 9(b) shows that the morphology of the *γ*′ phase in the alloy has been changed, which indicated that the large cubical *γ*′ has been repeatedly cut by dislocations. Thereby, there seem many fine particles on the surface of large *γ*′. These morphologies were the results of dislocation cutting the large *γ*′. So the *γ*′ phase has a certain strengthening role in the alloy. In Figure 9(c), the dislocation glide in the *γ* phase in the 5Ni alloy and the morphologies of the *γ*′ phase could not be seen under the effect of temperature and stress, as shown in Figure 9(d). In this manner, the *γ*′ phase cannot play a remarkable strengthening role. Therefore, the high-temperature compression performance of the 5Ni alloy is poor.  Figure 9(e) shows that not only do a large number of dislocations exist in the *γ*′ phase of the 10Ni alloy but also some stacking faults appear in the *γ* phase matrix channels. But the shape of the *γ*′ phase changed, and the size is rather small compared to that in the 0Ni alloy. The volume faction of the *γ*′ phase dropped remarkably compared to the alloy prior to the compression test, as shown in Figure 9(f). For the Co-30Ni alloy that has a high content of Ni, the dislocations easily shear the *γ*′ precipitates [50]. The massive dislocation tangles around the *γ*′ precipitates indicating that the *γ*′ is effective in retarding dislocation movement. The surface of retained *γ*′ is smooth, indicating that the *γ*′ particle is hard to be cut by dislocation. And although the *γ*′ phase of the alloy becomes significantly smaller and the volume fraction dropped, the *γ*′ phase in the 10Ni alloy still can play a key strengthening role. The strengthening effect of *γ*′ in the 10Ni alloy is better than that in the 0Ni alloy which may result from the higher Ti concentration induced by more Ni alloying, which is illustrated in Figure 5.

## 4. Conclusions

In conclusion, the effects of Ni on microstructure, elemental partition behavior, phase transition temperature, lattice misfit between *γ* and *γ*′ phases, and mechanical properties of the 0Ni, 5Ni, and 10Ni alloys were investigated. The lattice misfit between *γ* and *γ*′ phases of the alloys decreases from 0.74% to 0.61% as the Ni contents increase from 0 to 10%. There is a dual-size *γ*′ precipitate phase in the alloys. The larger precipitate was cuboidal in shape, and the average sizes of the *γ*′ phase were measured to be 312.10 nm, 112.86 nm, and 141.84 nm for the 0Ni, 5Ni, and 10Ni, respectively. The smaller *γ*′ precipitates were spherical particles with an average size of 18.09 nm, 16.95 nm, and 17.89 nm for the 0Ni, 5Ni, and 10Ni, respectively. Ti, V, and Ni exhibit a slight tendency to partition into the *γ*′ phase, while Co shows a slight tendency to partition into the *γ* phase. The solvus temperatures of the *γ*′ phase were measured to be 1167°C, 1114°C, and 1108°C for the 0Ni, 5Ni, and 10Ni alloys, respectively, by using differential scanning calorimetry (DSC), and the solvus temperature of the *γ*′ phase decreases as the Ni content increases. The yield strength of the 0Ni, 5Ni, and 10Ni alloys is about 176 MPa, 105 MPa, and 219 MPa, respectively. And ultimate strength of the 0Ni, 5Ni, and 10Ni alloys is about 235 MPa, 131 MPa, and 240 MPa, respectively. The yield strength and the ultimate strength of the 10Ni alloy are the highest. The *γ*′ phase can play a certain strengthening role in the 0Ni and the 10Ni alloy; thus, the high-temperature compressive strength of the 0Ni and 10Ni alloys is relatively high. However, the *γ*′ phase of the 5Ni alloy is no longer visible; thereby, it cannot play a good strengthening role; consequently, its strength is the lowest.

## Figures and Tables

**Table 1:** The nominal composition of the experimental alloy (at. %).

Alloy	Co	Ti	V	Ni
Co-8Ti-11V	Bal.	8	11	0
Co-8Ti-11V-5Ni	Bal.	8	11	5
Co-8Ti-11V-10Ni	Bal.	8	11	10

**Table 2:** Lattice constants and lattice misfit between *γ* and *γ*′ phases of alloys with different Ni contents.

Alloys	*a*_*γ*′_ (nm)	*a*_*γ*_ (nm)	*δ* (%)
Co-8Ti-11V	0.3611	0.3584	0.74
Co-8Ti-11V-5Ni	0.3607	0.3583	0.68
Co-8Ti-11V-10Ni	0.3593	0.3571	0.61

**Table 3:** Equilibrium compositions of the alloy with different Ni contents.

Alloys		Co (%)	Ti (%)	V (%)	Ni (%)
Co-8Ti-11V	*γ*	87.97	3.30	8.73	—
	*γ*′	78.95	8.11	12.95	—
Co-8Ti-11V-5Ni	*γ*	82.72	2.82	10.17	4.29
	*γ*′	75.36	6.23	13.48	4.93
Co-8Ti-11V-10Ni	*γ*	77.22	2.49	9.87	10.42
	*γ*′	68.31	6.47	13.56	11.66

## Data Availability

The PDF data used to support the findings of this study have been deposited in PDF 2002 card no. 23-0938.

## References

[1] Nishizawa T., Ishida K. (1983). The Co-Ni (cobalt-nickel) system. *Bulletin of Alloy Phase Diagrams*.

[2] Swartzendruber L., Itkin V., Alcock C. (1991). The Fe-Ni (iron-nickel) system. *Journal of Phase Equilibria*.

[3] Huang W., Chang Y. A. (1991). A thermodynamic analysis of the Ni-Al system. *Intermetallics*.

[4] Pollock T. M. (2016). Alloy design for aircraft engines. *Natural Materials*.

[5] Suzuki A., DeNolf G. C., Pollock T. M. (2007). Flow stress anomalies in *γ*/*γ*′ two-phase Co-Al-W-base alloys. *Scripta Materialia*.

[6] Pollock T. M., Dibbern J., Tsunekane M., Zhu J., Suzuki A. (2010). New Co-based *γ*-*γ*′ high-temperature alloys. *JOM*.

[7] Lass E. A., Williams M. E., Campbell C. E., Moon K. M., Kattner U. R. (2014). *γ*′ phase stability and phase equilibrium in ternary Co-Al-W at 900°C. *Journal of Phase Equilibria and Diffusion*.

[8] Lass E. A., Grist R. D., Williams M. E. (2016). Phase equilibria and microstructural evolution in ternary Co-Al-W between 750 and 1100°C. *Journal of Phase Equilibria and Diffusion*.

[9] Yan H. Y., Vorontsov V. A., Dye D. (2014). Alloying effects in polycrystalline *γ*′ strengthened Co-Al-W base alloys. *Intermetallics*.

[10] Chinen H., Sato J., Omori T. (2007). New ternary compound Co_3_(Ge, W) with L1_2_ structure. *Scripta Materialia*.

[11] Atas M. S., Yildirim M. (2020). Morphological development, coarsening, and oxidation behavior of Ni-Al-Nb superalloys. *Journal of Materials Engineering and Performance*.

[12] Plotnikov Elizaveta Y., Mao Z., Sung-Il B. (2019). A correlative four-dimensional study of phase-separation at the subnanoscale to nanoscale of a Ni Al alloy. *Acta Materialia*.

[13] Atas M. S., Yildirim M. (2019). Temporal evolution, coarsening behavior and oxidation resistance of Ni–15Al superalloy. *Journal of Alloys and Compounds*.

[14] Sato J., Omori T., Oikawa K., Ohnuma I., Kainuma R., Ishida K. (2006). Cobalt-base high-temperature alloys. *Science*.

[15] Kobayashi S., Tsukamoto Y., Takasugi T. (2009). Determination of phase equilibria in the Co-rich Co-Al-W ternary system with a diffusion-couple technique. *Intermetallics*.

[16] Shinagawa K., Omori T., Sato J. (2008). Phase equilibria and microstructure on *γ*′ phase in Co-Ni-Al-W system. *Materials Transactions*.

[17] Povstugar I., Choi P. P., Neumeier S. (2014). Elemental partitioning and mechanical properties of Ti- and Ta-containing Co-Al-W-base superalloys studied by atom probe tomography and nanoindentation. *Acta Materialia*.

[18] Shinagawa K., Chinen H., Omori T., Oikawa K., Ohnuma I., Kainuma R. (2014). Phase equilibria and thermodynamic calculation of the Co-Ta binary system. *Intermetallics*.

[19] Ooshima M., Tanaka K., Okamoto N. L., Kishida K., Inui H. (2010). Effects of quaternary alloying elements on the *γ*′ solvus temperature of Co–Al–W based alloys with fcc/L1_2_ two-phase microstructures. *Journal of Alloys and Compounds*.

[20] Bocchini P. J., Sudbrack C. K., Noebe R. D., Dunand D. C., Seidman D. N. (2017). Microstructural and creep properties of boron- and zirconium-containing cobalt-based superalloys. *Materials Science and Engineering A*.

[21] Bocchini P. J., Lass E. A., Moon K. W. (2013). Atom-probe tomographic study of *γ*/*γ*′ interfaces and compositions in an aged Co-Al-W superalloy. *Scripta Materialia*.

[22] Bocchini P. J., Sudbrack C. K., Sauza D. J., Noebe R. D., Seidman D. N., Dunand D. C. (2017). Effect of tungsten concentration on microstructures of Co-10Ni-6Al-(0,2,4,6)W-6Ti (at%) cobalt-based superalloys. *Materials Science and Engineering A*.

[23] Sauza D. J., Bocchini P. J., Dunand D. C., Seidman D. N. (2016). Influence of ruthenium on microstructural evolution in a model Co-Al-W superalloy. *Acta Materialia*.

[24] Vorontsov V. A., Barnard J. S., Rahman K. M., Yan H. Y., Midgley P. A., Dye D. (2016). Coarsening behaviour and interfacial structure of *γ*′ precipitates in Co-Al-W based superalloys. *Acta Materialia*.

[25] Makineni S. K., Nithin B., Chattopadhyay K. (2015). A new tungsten-free *γ*-*γ*′ Co-Al-Mo-Nb-based superalloy. *Scripta Materialia*.

[26] Makineni S. K., Nithin B., Palanisamy D., Chattopadhyay K. (2016). Phase evolution and crystallography of precipitates during decomposition of new “tungsten-free” Co(Ni)–Mo–Al–Nb *γ*-*γ*′ superalloys at elevated temperatures. *Journal of Materials Science*.

[27] Makineni S. K., Samanta A., Rojhirunsakool T. (2015). A new class of high strength high temperature cobalt based *γ*-*γ*′ Co-Mo-Al alloys stabilized with Ta addition. *Acta Materialia*.

[28] Makineni S. K., Nithin B., Chattopadhyay K. (2015). Synthesis of a new tungsten-free *γ*-*γ*′ cobalt-based superalloy by tuning alloying additions. *Acta Materialia*.

[29] Aoki Y., Echigoya J. (1985). Phase separation in an 18.2 at% V-Co alloy annealed at 1073 K. *Scripta Metallurgica*.

[30] Viatour P., Drapier J. M., Coutsouradis D. (1973). Stability of the gamma prime Co_3_Ti compound in simple and complex Co alloys. *Cobalt*.

[31] Zhou G. J., Tang J. G., Zhou Y., An W. K., Cai A. H. (2014). Phase equilibria in the Co-Ti-V system at 873 K. *Journal of Alloys and Compounds*.

[32] Tirado F. L. R., Toinin J. P., Dunand D. C. (2018). *γ*+*γ*′ microstructures in the Co-Ta-V and Co-Nb-V ternary systems. *Acta Materialia*.

[33] Davydov A. V., Kattner U. R., Jossel D. (2001). Determination of the Co-Ti congruent melting point and thermodynamic reassessment of the Co-Ti system. *Metallurgical and Materials Transactions A: Physical Metallurgy and Materials Science*.

[34] Zenk C. H., Povstugar I., Li R. (2017). A novel type of Co-Ti-Cr-base *γ*/*γ*′ superalloys with low mass density. *Acta Materialia*.

[35] Ruan J. J., Wang C. P., Zhao C. C., Yang S. Y., Yang T., Liu X. J. (2014). Experimental investigation of phase equilibria and microstructure in the Co-Ti-V ternary system. *Intermetallics*.

[36] Ruan J. J., Liu X. J., Yang S. Y. (2018). Novel Co-Ti-V-base superalloys reinforced by L1_2_-ordered *γ*′ phase. *Intermentallics*.

[37] Meher S., Nag S., Tiley J., Goel A., Banerjee R. (2013). Coarsening kinetics of *γ*′ precipitates in cobalt-base alloys. *Acta Materialia*.

[38] Pandey P., Sawant A. K., Nithin B. (2019). On the effect of Re addition on microstructural evolution of a CoNi-based superalloy. *Acta Materialia*.

[39] Wang W. Z., Jin T., Liu J. L., Sun X. F., Guan H. R., Hu Z. Q. (2008). Role of Re and Co on microstructures and *γ*′ coarsening in single crystal superalloys. *Materials Science and Engineering A*.

[40] Li L. L., Wang C. P., Chen Y. C. (2019). Effect of Re on microstructure and mechanical properties of *γ*/*γ*′ Co-Ti-based superalloys. *Intermetallics*.

[41] Takasugi T., Izumi O. (1985). High temperature strength and ductility of polycrystalline Co_3_Ti. *Acta Metallurgica*.

[42] Takasugi T., Izumi O. (1985). Recrystallization and grain growth of Co_3_Ti. *Acta Metallurgica*.

[43] Shinagawa K., Omori T., Oikawa K., Kainuma R., Ishida K. (2009). Ductility enhancement by boron addition in Co-Al-W high-temperature alloys. *Scripta Materialia*.

[44] Bocchini P. J., Sudbrack C. K., Noebe R. D., Dunand D. C., Seidman D. N. (2017). Effects of titanium substitutions for aluminum and tungsten in Co-10Ni-9Al-9W (at%) superalloys. *Materials Science and Engineering*.

[45] Nithin B., Samanta A., Makineni S. K. (2017). Effect of Cr addition on *γ*-*γ*′ cobalt-based Co-Mo-Al-Ta class of superalloys: a combined experimental and computational study. *Journal of Materials Science*.

[46] Omori T., Oikawa K., Sato J. (2013). Partition behavior of alloying elements and phase transformation temperatures in Co-Al-W-base quaternary systems. *Intermetallics*.

[47] Jahangiri M. R., Boutorabi S. M. A., Arabi H. (2012). Study on incipient melting in cast Ni base IN939 superalloy during solution annealing and its effect on hot workability. *Materials Science and Technology*.

[48] Zhang J. X., Harada H., Koizumi Y., Kobayashi T. (2010). Dislocation motion in the early stages of high-temperature low-stress creep in a single-crystal superalloy with a small lattice misfit. *Journal of Materials Science*.

[49] Chen J. Y., Zhao B., Feng Q., Cao L. M., Sun Z. Q. (2010). Effects of Ru and Cr on *γ*/*γ*′ microstructural evolution of Ni-based single crystal superalloys during heat treatment. *Acta Metallurgica Sinica*.

[50] Qu S. S., Li Y. J., He M. L. (2019). Microstructural evolution and compression property of a novel *γ*′-strengthened directionally solidified CoNi-base superalloy. *Materials Science and Engineering A*.

